# RETRACTED: Ibrahim et al. Potent Quinoline-Containing Combretastatin A-4 Analogues: Design, Synthesis, Antiproliferative, and Anti-Tubulin Activity. *Pharmaceuticals* 2020, *13*, 393

**DOI:** 10.3390/ph19081174

**Published:** 2026-07-27

**Authors:** Tarek S. Ibrahim, Mohamed M. Hawwas, Azizah M. Malebari, Ehab S. Taher, Abdelsattar M. Omar, Niamh M. O’Boyle, Eavan McLoughlin, Zakaria K. Abdel-Samii, Yaseen A. M. M. Elshaier

**Affiliations:** 1 Department of Pharmaceutical Chemistry, Faculty of Pharmacy, King Abdulaziz University, Jeddah 21589, Saudi Arabia; amelibary@kau.edu.sa (A.M.M.); asmansour@kau.edu.sa (A.M.O.); 2 Department of Pharmaceutical Organic Chemistry, Faculty of Pharmacy, Zagazig University, Zagazig 44519, Egypt; zakariaabdelsamii@yahoo.com; 3 Department of Pharmaceutical Organic Chemistry, Faculty of Pharmacy, Al-Azhar University, Assiut 71524, Egypt; mohhawwas@yahoo.com (M.M.H.); ehabtaher@azhar.edu.eg (E.S.T.); 4 Department of Pharmaceutical Chemistry, Faculty of Pharmacy, Al-Azhar University, Cairo 11884, Egypt; 5 School of Pharmacy and Pharmaceutical Sciences, Trinity College Dublin, Trinity Biomedical Sciences Institute, 152-160 Pearse Street, Dublin 2, Ireland; niamh.oboyle@tcd.ie (N.M.O.); mclougea@tcd.ie (E.M.); 6 Department of Organic and Medicinal Chemistry, Faculty of Pharmacy, University of Sadat City, Sadat City 32958, Egypt; yaseenorganic@yahoo.com

The journal retracts the article titled, “Potent Quinoline-Containing Combretastatin A-4 Analogues: Design, Synthesis, Antiproliferative, and Anti-Tubulin Activity” [1], cited above.

Following publication, concerns were brought to the attention of the Editorial Office regarding the integrity of the images presented in this publication [1].

In accordance with the journal’s standard procedures, the Editorial Office and Editorial Board conducted an investigation that identified indications of inappropriate image editing of Figures 4A and 5A published in [1]. While the authors fully collaborated within this process, the supporting material and explanations provided did not meet Editorial Board expectations in order to fully resolve the issues raised. As a consequence, the Editorial Bord lost confidence in the validity of images and has decided to retract this publication in accordance with MDPI’s retraction policy.

This retraction was approved by the Editor-in-Chief of the journal *Pharmaceuticals* and invites the authors to revise and resubmit the manuscript for consideration, with the inclusion of a new data set and full transparency, as per MDPIs’ standard editorial process.

The authors have been informed of this retraction and have indicated their disagreement with this decision.
